# Modulation of Alpha-Synuclein Aggregation by Dopamine Analogs

**DOI:** 10.1371/journal.pone.0009234

**Published:** 2010-02-16

**Authors:** Diane Latawiec, Fernando Herrera, Alpan Bek, Valeria Losasso, Michela Candotti, Federico Benetti, Elvio Carlino, Agata Kranjc, Marco Lazzarino, Stefano Gustincich, Paolo Carloni, Giuseppe Legname

**Affiliations:** 1 Department of Neurobiology, Scuola Internazionale Superiore di Studi Avanzati–International School for Advanced Studies (SISSA-ISAS), Trieste, Italy; 2 Department of Statistical and Biological Physics, Scuola Internazionale Superiore di Studi Avanzati–International School for Advanced Studies (SISSA-ISAS), Trieste, Italy; 3 Italian Institute of Technology–SISSA Unit, Trieste, Italy; 4 Consorzio per il Centro di Biomedicina Molecolare–Center for Molecular Biomedicine (CBM Scrl), Trieste, Italy; 5 TASC-INFM National Laboratory, Trieste, Italy; 6 ELETTRA Laboratory, Sincrotrone Trieste S.C.p.A, Trieste, Italy; Brigham and Women's Hospital/Harvard Medical School, United States of America

## Abstract

The action of dopamine on the aggregation of the unstructured alpha-synuclein (α-syn) protein may be linked to the pathogenesis of Parkinson's disease. Dopamine and its oxidation derivatives may inhibit α-syn aggregation by non-covalent binding. Exploiting this fact, we applied an integrated computational and experimental approach to find alternative ligands that might modulate the fibrillization of α-syn. Ligands structurally and electrostatically similar to dopamine were screened from an established library. Five analogs were selected for *in vitro* experimentation from the similarity ranked list of analogs. Molecular dynamics simulations showed they were, like dopamine, binding non-covalently to α-syn and, although much weaker than dopamine, they shared some of its binding properties. *In vitro* fibrillization assays were performed on these five dopamine analogs. Consistent with our predictions, analyses by atomic force and transmission electron microscopy revealed that all of the selected ligands affected the aggregation process, albeit to a varying and lesser extent than dopamine, used as the control ligand. The *in silico/in vitro* approach presented here emerges as a possible strategy for identifying ligands interfering with such a complex process as the fibrillization of an unstructured protein.

## Introduction

**Figure 1:**
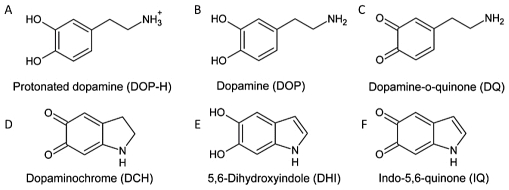
Dopamine and the oxidation derivatives known to interact with α-syn. (A) Protonated Dopamine (DOP-H), (B) Dopamine (DOP), (C) Dopamine-o-quinone (DQ), (D) Dopaminochrome (DCH), (E) 5,6-Dihydroxyindole (DHI), (F) Indol-5,6-quinone (IQ).

Parkinson's disease (PD) is a neurodegenerative movement disorder, affecting an estimated four million people worldwide [1], [2]. It is characterized by the loss of the neuromelanin expressing dopamine (DOP) neurons in the *substantia nigra pars compacta* and the deposition of Lewy bodies in many of the cells remaining in this region [3], [4], [5], [6], [7], [8], [9]. The major components of the Lewy bodies are fibrillar aggregates of the alpha-synuclein protein (α-syn) [6], [10], [11]. Thus, α-syn fibrillization and DOP metabolism are likely to be linked to PD pathogenesis [11], [12], [13], [14], [15], [16], [17], [18], [19], [20], [21]. DOP and some derivatives (Figure 1), which may be present in oxidizing conditions, form non-covalent and/or covalent adducts with α-syn [21], [22]. These molecules inhibit the conversion of α-syn to mature fibrils, promoting instead, accumulation of oligomeric (or protofibril) forms [23], [24], [25], [26], [27].

Recently, molecular dynamics (MD) simulations based on α-syn's nuclear magnetic resonance (NMR) structural ensemble [28] in combination with biophysical methods, led some of us to propose a structural basis for DOP non-covalent inhibition of α-syn fibrillization [29]. This may be caused, at least in part, by 1) the formation of nonspecific hydrophobic contacts between DOP and its oxidation derivatives with the C-terminal; this includes the ^125^YEMPS^129^ region, as in agreement with experimental evidence [23], [28] and 2) long-range electrostatic interactions with residues in the NAC region which are involved in the fibrillization process [29].

**Figure 2:**
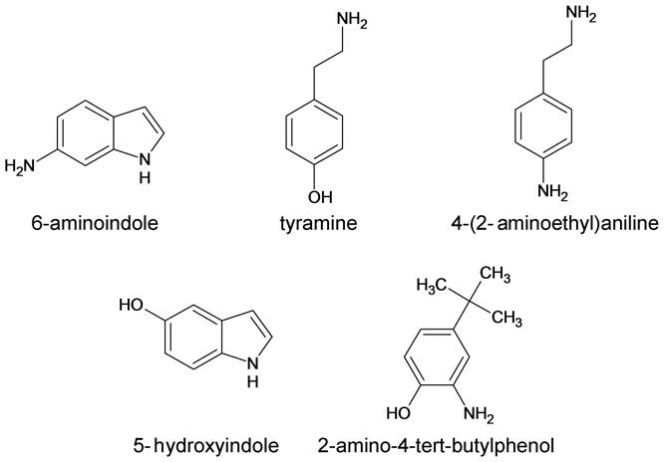
Chemical structures of the five DOP analogs chosen for the *in silico/in vitro* analysis.

Molecules structurally and electrostatically similar to a given ligand might provide similar structure/activity relationships [30]. We screened ligands structurally and electrostatically similar to DOP (Figure 2) from the *ligand.info* meta-database [31]. The ability of these ligands to bind to α-syn was then explored by MD simulations. The ligands bound weaker to the protein than DOP. Consistently, high-resolution atomic force microscopy (AFM) and transmission electron microscopy (TEM) data showed that the ligands affected α-syn fibril assembly, but to a lower extent than DOP. Remarkably, the best analogs revealed the most inhibitory effects upon protein aggregation in terms of fibril length and quantity.

## Results

### Dopamine Mimics: Identification and Binding to α-Syn

We screened seventy molecules of the *ligand.info* database [31] with the largest similarity with DOP and its oxidation derivatives (Table S1). The similarity was quantified according to the Tanimoto's equations [32]. The ligands were docked onto the six structural representatives of human α-syn in aqueous solution. The procedure was identical to that of previously reported [29], except that a refined set of structures of α-syn were used (see Material and Methods). The resulting complexes were ranked in terms of the number of contacts with the ^125^YEMPS^129^ region known-to-be targeted by DOP [24], [26], [29]. Such ranking was used only as a means to fast screen the ligands, and not to provide structural predictions. In fact, there are severe limitations of this procedure for an unstructured protein, as discussed by Slutzki et al [33] Five commercially available ligands chosen randomly from Table S1 and forming top ranking adducts (Figure 2), underwent 36 ns MD simulations in water solution (Table S2). The same procedure was used for DOP and its derivatives (Figure 1).

DOP and its oxidation derivatives bound for 69% or more of their time to α-syn (the criteria used to identify bound complexes are described in Materials and Methods) and bound preferentially to the target ^125^YEMPS^129^ region, similarly to previous MD simulations [29]. As found previously [29], they did not form specific interactions with the target region (Table S2). Moreover, they interacted with one or both negative residues (E83 and E61) of the NAC region (Table 1), a region known to be involved in the fibrillization of the protein. DOP-E83 interaction might play a role in the observed inhibition of fibrillization by DOP, as suggested experimentally [29].

**Table 1:** **α-syn binding of ligands in figures 1, 2 in aqueous solution observed in MD simulations**. DOP and its oxidation derivatives abbreviations are explained in detail in figure 1. Column titles from left to right: (i) ligand name (ii) percentage of time in which the ligands are bound to α-syn and (iii) to the ^125^YEMPS^129^ ‘target’ region, (iv-v) stabilizing electrostatic interaction energies between the ligands and E83 and E61, two negatively charged residues of the NAC region. Energy values of the force field are very approximate^1^ and do not take into account the screening of the solvent. They should be taken here only for qualitative comparisons. Here they are normalized with respect to the most negative interaction energy between the neutral ligands and the two negative residues (-2.8 kcal/mol, relative to the interaction between IQ and E83). The DOPH/E61 energy turns out to be very large in absolute value because the ligand is charged, in contrast to all the others.

Ligand	% protein	% target	Point Charge Model Av ± Std (E83)	Point Charge Model Av ± Std (E61)
DOPH	70	32	0.0	−14.9
DOP	69	46	0.0	−0.7
DQ	49	28	−0.3	−0.3
DCH	72	32	−0.8	−0.5
DHI	91	16	0.0	0.0
IQ	62	32	−1.0	−0.3
6-aminoindole	68	23	0.0	0.0
Tyramine	48	19	0.0	0.1
4-(2-aminoethyl) aniline	39	9	0.0	-0.2
5-hydroxyindole	70	23	-0.9	0.0
2-amino-4-tert-butylphenol	62	19	0.0	0.0

1 L. Guidoni, V. Torre and P. Carloni, FEBS Letters 477 (2000) 37-42.

Three of the screened ligands (6-aminoindole, 5-hydroxyindole, 2-amino-4-tert-butylphenol) interacted significantly with α-syn (and in particular with the ^125^YEMPS^129^ region), although to a lesser extent than most dopamine derivatives (Table 1). In addition, they formed much weaker stabilizing electrostatic interactions with E61 and/or E83 (Table 1).

We have also studied several binding regions of ligands other than the target region. The results are provided in Table S3, where the contacts are listed for those adducts in which the ligands bind to regions other than the target region for more than 50% of their time.

The other two ligands, tyramine and 4-(2-aminoethylaniline), bound much less to α-syn (albeit still interacting with the ^125^YEMPS^129^ region), with almost no stabilizing interactions with the two residues (E61 and E83) of NAC region.

As in the case of DOP, all the ligands did not form specific interactions with any residues in the ^125^YEMPS^129^ region (Table S2).

Based on these results, we propose that: (i) the DOP mimics may interfere with the fibrillization of α-syn, although to a lesser extent than DOP and (ii) the ligands, that show the strongest binding to α-syn, i.e 6-aminoindole, and 5-hydroxyindole, and 2-amino-4-tert-butylphenol may have the strongest effect on the fibrillization of α-syn. We next proceeded to perform *in vitro* assays to test these two predictions.

### *In Vitro* Fibrillization Assays

Human α-syn was produced and purified as described previously [34], with some modifications. Protein analysis by SDS-PAGE resulted in a single band showing a molecular weight (MW) of ≈14 KDa. The exact MW, as determined by mass spectrometry, was 14459.40±0.43 Da, and far-UV circular dichroism (CD) measurements revealed a randomly coiled secondary structure.

**Figure 3:**
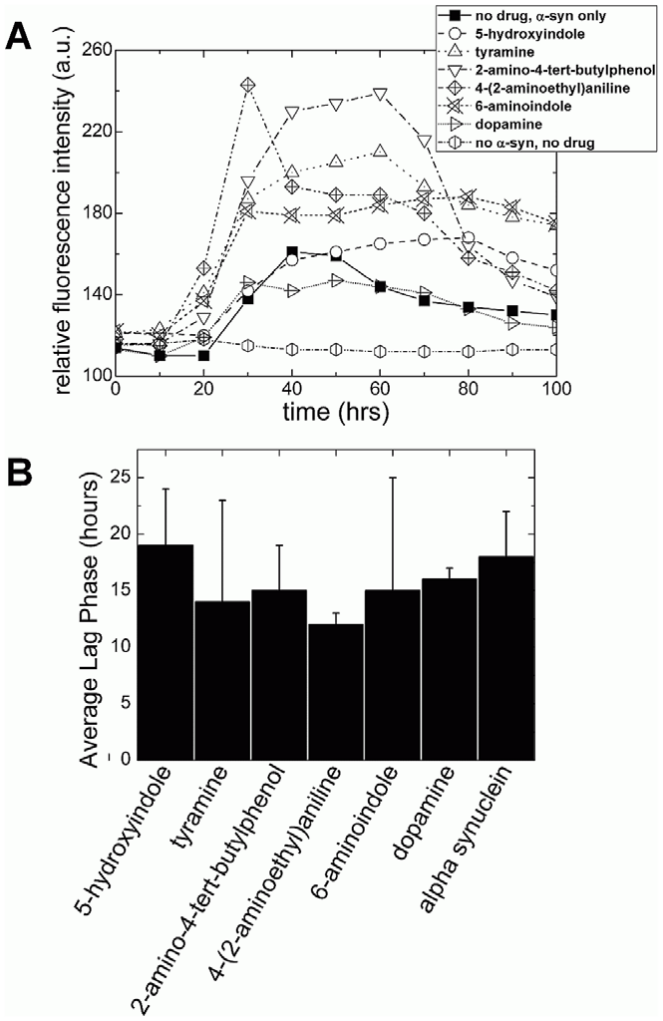
The kinetics of α-syn fibrillization with the DOP analogs in comparison to α-syn alone. (A) Kinetics curves of α-syn fibrillization in the presence of the test ligands. The fibrillization buffer (i.e, no protein) was assayed in the absence of both α-syn and the test ligands. All curves represent the mean kinetics output from at least 3 measurements. (B) Bar chart statistics displays no significant difference between the lag phases.

Human α-syn was then used in the amyloid fibrillization experiments in the presence and absence of the test ligands. For each assay run, a concentration of 100 µM of α-syn was used for the synthesis of the fibrils. All ligands were added together with the α-syn in an equimolar concentration at the start of the assay run. Due to its known inhibitory effect on α-synuclein fibrilization, dopamine was used as the control ligand [26]. Fibril formation was achieved using a continuous *in vitro* fibrillization assay, with amyloid fibril formation monitored by the dye, thioflavin T (ThT). Each assay was always run in triplicate. The assays ran for 100 hours, continuously, under a controlled temperature of 37°C. At the end of the assay, each sample revealed a kinetic curve suggestive of amyloid fibril formation [35], [36], [37] comprising of a lag phase, an exponential growth phase and an end plateau (Figure 3A), which is typical of a nucleated polymerization type process [38]. Moreover, there appeared to be no observable significant differences in the average lag phase time between all ligands (including dopamine) and α-syn alone (Figure 3B).

For all *in vitro* assays, the ligands and the control ligand DOP were dissolved in a concentration of 0.1% dimethylsulfoxide (DMSO). This concentration had been determined empirically by a series of preliminary experiments set out to elucidate the concentration where DMSO had no effect, upon both the lag phase and the assembly of fibrils (see Figure S1, S2 and S3).

**Figure 4:**
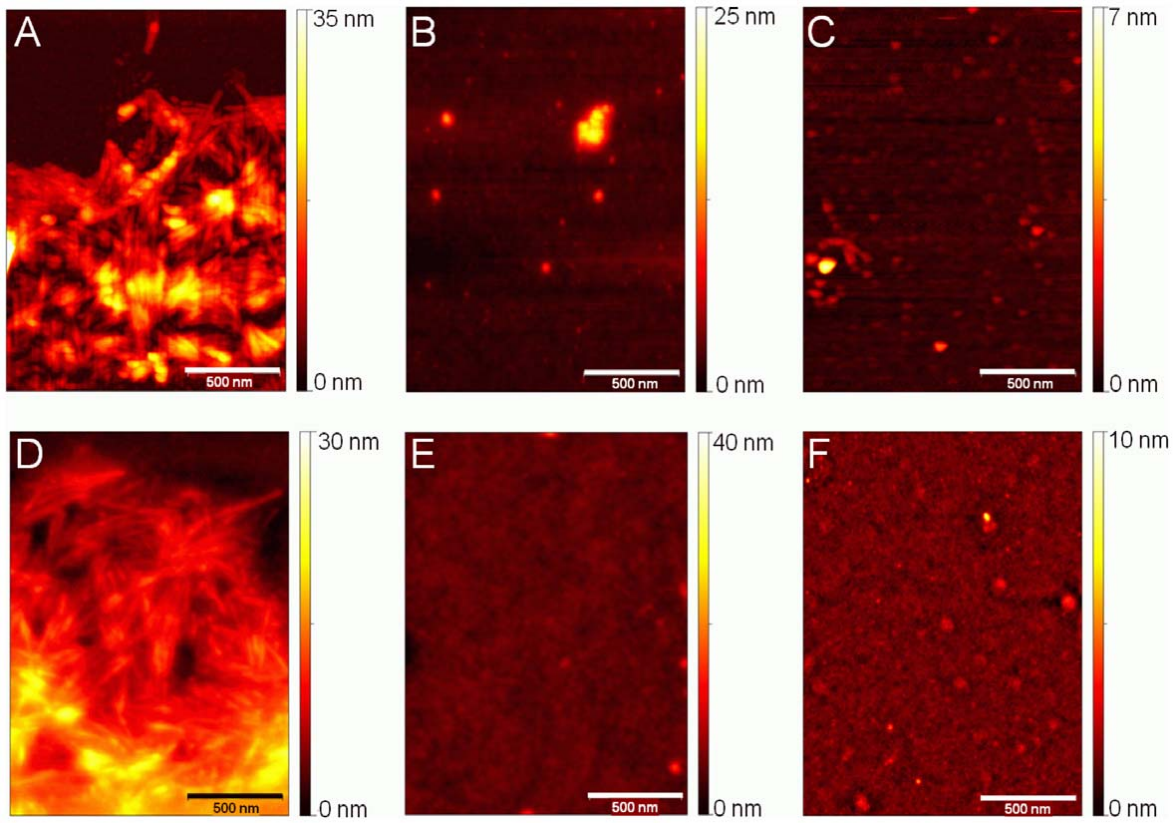
AFM analysis of α-syn aggregation in the presence or absence of both ThT and DOP. AFM height images were acquired from assay end products, which had been deposited onto freshly cleaved mica surfaces after 100 hours of incubation. The presence or absence of ThT revealed no observable effect upon either the formation or morphology of α-syn fibrils when assay was run with α syn alone, (A) +ThT, (D)–ThT. The inhibitory effect of DOP on the α syn fibrillization was clearly observed when the assay was run in the presence of DOP. (B, C, E, F). Moreover, the presence (B, C) or absence (E, F) of ThT did not show any effect upon the inhibitory action of DOP on α-syn fibrillization.

The continuous presence of ThT in the assay was assessed by AFM, to determine whether this could have an effect upon the aggregation of the protein and/or the binding of a ligand to the α-syn. As DOP has previously been shown by AFM to inhibit the aggregation of α-syn [26], the reaction of DOP with α-syn protein in equimolar concentration in the assay, was assessed by AFM, both in the presence and absence of the ThT dye. Neither the presence nor the absence of ThT appeared to have any effect on the inhibitory effect of DOP on the aggregation of α-syn (Figure 4).

### AFM and TEM Analysis of the Ligands on the Aggregation of α-Syn

**Figure 5:**
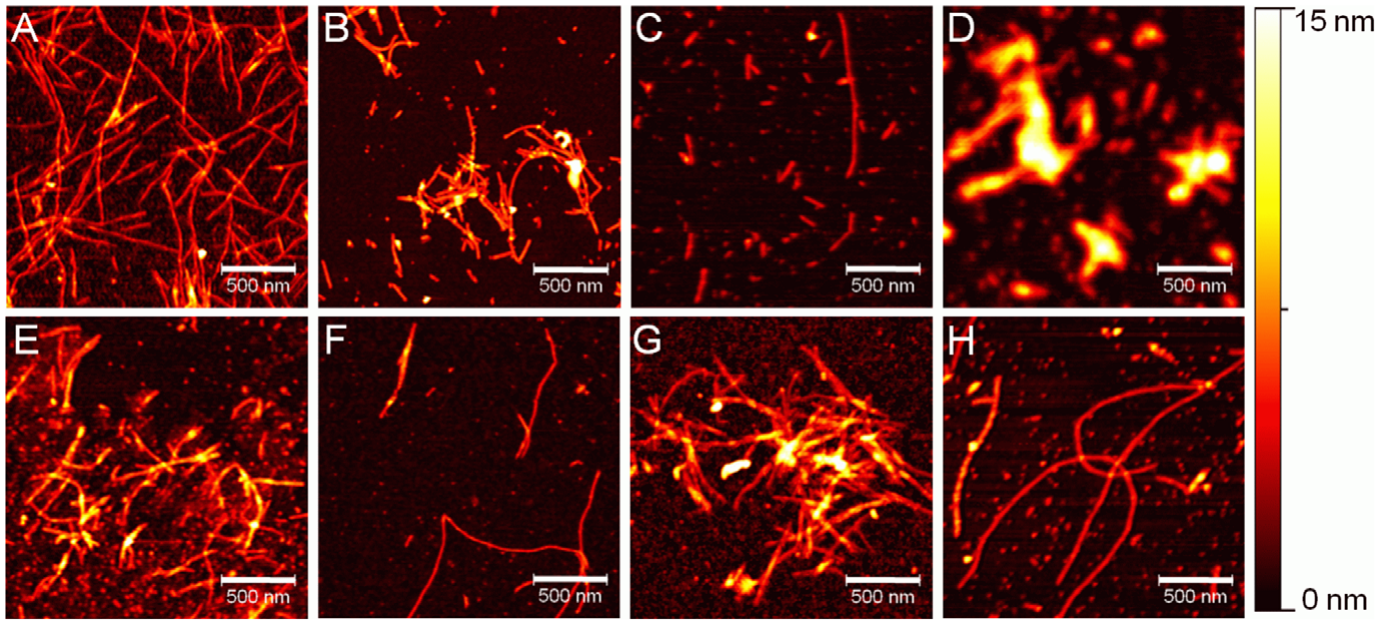
AFM analysis of α-syn aggregation in presence of test ligands as DOP analogs and alone. AFM height images were acquired from assay end products, which had been deposited onto freshly cleaved mica surfaces after 100 hours of incubation. All images are displayed using the same color scale as shown on the right hand side. (A–B) 5-hydroxyindole; (C) 4-(2-aminoethyl)aniline; (D-E) 6-aminoindole; (F) 2-amino-4-tert-butylphenol; (G) tyramine; (H) α-syn only.

Samples were analyzed by both AFM and TEM. In order to detect any distinctive effect upon the assembly of the fibrils, we developed a detailed classification of the fibrillar structures that had been formed. Based on AFM analysis, fibrillar structures were classified as follows: mature fibrils (>0.75 µm in length), intermediate fibrils (0.5–0.75 µm), short fibrils/fragments (or protofibrils) (<0.5 µm). Qualitatively, AFM analysis revealed some differences in terms of α-syn fibril assembly in the presence of the ligands (Figure 5*A–G*).

**Figure 6:**
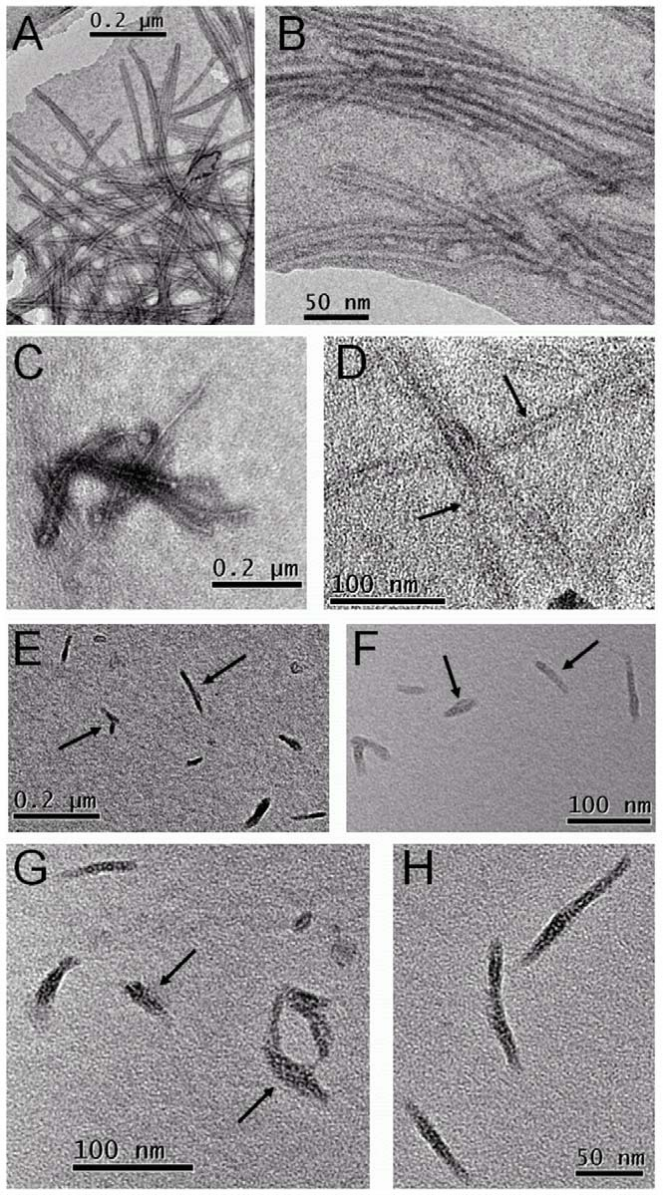
TEM micrographs showing the effect of the presence of 6-aminoindole or 5-hydroxindole on the aggregation of α-syn. Whilst both samples revealed similar kinetic data, typical of a nucleation/polymerization process, microscopy revealed contrasting data. (A and B) α-syn fibrils formed in the absence of any ligands, (C and D) α-syn fibrils formed in the presence of 5-hydroxyindole and (E–H) 6-aminoindole. Arrows indicate fibrillar structures of varying size.

The α-syn fibrils in the absence of any of the ligands consistently revealed predominantly ‘mature’ fibrils (≈>0.75 µm in length) with an average width of ≈9.2 nm ±2.2 nm on TEM micrographs (107 fibrils) (Figure 4*A* and *D*, Figure 5*H* and Figure 6*A* and *B*, and Figure S2 and Figure S3), in agreement with previous reports [39], [40], [41]. Moreover, they frequently appeared in clusters (Figure 4*A* and *D*, Figure 6*A* and *B*, Figure S2 and Figure S3). In addition, round spherical structures (possibly oligomers) were observed (Figure 5*H* and Figure S2, Figure S3).

In the presence of the test ligands, in an equimolar concentration with α-syn, the following observations were made. Overall, whilst all ligands showed no complete inhibition on the assembly of α-syn fibrils (Figure 5) compared to the control ligand DOP (Figure 4), differences did appear in both fibril size and distribution of particle aggregates (Figure 5*A–G*) when compared to α-syn alone (Figure 4*A* and *D*, Figure 5*H*, Figure S2), which consistently revealed mostly mature fibrils. DOP revealed no fibrils, and only spherical structures (Figure 4*B, C, E* and *F*), whereas 5-hydroxyindole and 6-aminoindole revealed predominantly fibrils of an intermediate and short size (Figure 5*A, B, D, E*). Tyramine and 2-amino-4-tert-butylphenol revealed a mixture of both mature and intermediate fibrils (Figure 5*F* and *G*), whereas 4-(2-aminoethylaniline) revealed a mixture of all fibril sizes (Figure 5*C*). The ligand 6-aminoindole appeared overall to show the greatest effect on the assembly of α-syn fibrils. As MD simulations predicted that this ligand and 5-hydroxyindole should have the strongest binding to α-syn (Table 1), these two ligands were chosen for a more detailed analysis by higher resolution with TEM.

By TEM, α-syn fibrils alone were generally observed in clusters and longer than 0.75 µm in length (Figure 6A–B). In comparison, 6-aminoindole revealed individual structures, orientated in a fibrillar form (Figure 6*E–H*), which at high magnification suggested that these ‘fibrils’ could still be at an early/intermediate stage of the fibrillization process. The ligand 5-hydroxyindole in comparison showed clusters of α-syn fibrils, albeit shorter than α-syn fibrils in the absence of this ligand (Figure 6*C–D* and Figure 6*A–B*, respectively).

## Discussion

The loss of dopaminergic neurons in the *substantia nigra* and the presence of α-syn containing Lewy bodies are the classical diagnostic markers of PD. DOP has been shown to inhibit α-syn aggregation by binding to the α-syn protein [21], [22], [23], [24], [25], [26], [27], [29]. Thus, DOP mimics might have some effect on the aggregation of the protein.

We analyzed this issue using a combined *in silico* and *in vitro* approach. Ligand screening and molecular docking allowed us to identify the five commercially available ligands used in this study, which are structurally and electrostatically similar to DOP (Figure 2). Thus, we predicted that these ligands might have a weaker inhibitory effect on fibrillization than DOP.

To test our predictions, we developed a continuous *in vitro* fibrillization assay. We observed the kinetics of the fibrillization process and analyzed the reaction end products by AFM to obtain spatially resolved information of their morphology. AFM revealed that the ligands caused some inhibitory effect, albeit weaker than that of DOP (Figure 5 and Figure 4 respectively). Moreover, it was clearly apparent that the fibrillar structures varied in terms of their length and in the population distribution of the structures for each test ligand (Figure 5), although the kinetic data were typical of an occurring nucleation/polymerization mechanism (Figure 3A). Likewise, the kinetics curves for each ligand did vary in terms of exponential growth time and plateau phases (Figure 3A). The ligand 6-aminoindole revealed the strongest inhibitory effect upon fibril formation, which was consistent with the fact that it binds most strongly (along with 5-hydroxindole) to the ^125^YEMPS^129^ ‘target’ region (Table 1). In comparison, 4-(2-aminoethyalinine) showed the weakest effect (Figure 5), consistent with our MD data (Table 1).

Whilst all the ligands used in our study share the same chemical scaffolding, we noticed (as revealed by both the kinetic output curve and AFM analysis) the strength of DOP in inhibiting α-syn fibril formation in comparison to the tested ligands, especially tyramine (Figure 3); which was consistent with our modeling (Table 1). Tyramine differs from DOP only by a hydrogen in place of a hydroxyl group. The removal of the polar OH function is associated with a loss of electrostatic interactions with the two negatively charged groups of the NAC region. We thus propose that the dihydroxyphenyl group (or 1,2 benzoquinone as in some dopamine derivatives, like dopaminochrome) may play an important role for binding to α-syn, possibly through long-range electrostatic interactions. Moreover, based on these considerations, we suggest that ligands with dihydroxyphenyl (or 1,2 benzoquinone) groups could be selected for further non-covalent binding assays.

Indoles are compounds that are known to interact with metal ions, which themselves are known to increase in PD brains [42]. Furthermore, metal ions are known to bind to, and facilitate, the aggregation of α-syn [42], [43]. Whilst we cannot exclude the presence of metal ions in our assay, we nevertheless ensured that the highest-grade analytical chemicals with the minimal presence of metal ions were always used.

Interestingly, the ultra-structural analysis of α-syn in the presence of 6-aminoindole not only showed fibrils more isolated and shorter than just α-syn alone (Figure 6*A–B*), but at a higher resolution it was possible to clearly identify that the process of ‘fibril’ developing into a mature fibril assembly had been affected by the ligand, when compared to α-syn alone (Figure 6*A* compared to 6*H*). Most importantly, the kinetic data for both α-syn with this ligand, and α-syn alone, revealed similar ThT fluorescence with the characteristic growth curve. Therefore, we only used ThT fluorescence as a monitor of amyloid fibril formation. A more detailed quantitative methodology of classification would aid to verify our observations. This is currently under development.

Our conclusions, drawn from the ultra-structural analysis of aggregated α-syn in the presence of 6-aminoindole, are further supported by a recent study by Tashiro *et al*. [44], which characterized over 76 hours of the fibrillization process of α-syn by electron microscopy and small angle x-ray scattering. The authors observed the progression of the formation of fibrils to a mature form using a discontinuous fibrillization assay. Whilst they clearly saw mature fibrils formed at 60 hours, our images at 100 hours for 6-aminoindole did not show this stage of formation (Fure 6*E–H*). Our TEM images for 6-aminoindole (Figure 6*E–H* and unpublished data) obtained at the end of the assay (*i.e.*: 100 hour) matched closest (if indeed that) with their images of the aggregation of α-syn at 33 hours, which, they speculated, could have been fibrils forming via a critical nucleus or soluble oligomers. Most importantly, their study clearly supports our implication that 6-aminoindole does indeed affect the nucleation/polymerization mechanism. Whilst the main aim of our study was to use *in vitro* assays to test our prediction of DOP mimics on α-syn, it is clear that a detailed high resolution study over more time points would lead to a deeper insight into the mechanism of the observed DOP mimics on the aggregation of α-syn.

In conclusion, the combined *in silico* and *in vitro* approach employed here is, to our knowledge, the first study where such a detailed approach has been used to predict and evaluate ligands that may affect the aggregation of α-syn by forming non-covalent interactions. Biocomputational methods screened and predicted ligands that could have some inhibitory effect on the aggregation of α-syn protein. Remarkably, *in vitro* assays in combination with high-resolution microscopy showed similar findings. The consistency between both approaches highlights the importance of a combined *in silico* and *in vitro* approach that could be used in predicting and developing new drugs and therapeutic strategies for PD.

## Materials and Methods

### Biocomputing of α-Syn Structures

A refined set of structures, obtained with an optimized protocol, was kindly provided by M. Vendruscolo (personal communication). A cluster analysis was performed exactly as previously reported [29]. Six representative structures were identified [29].

### Biocomputing of Ligands

(i) The structures of dopamine and its oxidation products (Figure 1 in [29]) were taken from a previous publication [23].

(ii) Ligands similar to DOP and to its oxidative derivatives (Figure 1) were identified by virtual screening of the ‘ligand.info’ database [31]. This database contains ≈1,160,000 ligands. Structural similarity was estimated by the Tanimoto's equation [32] using the ROCS algorithm in the OpenEye suite of programs (http://www.openeye.org). The 3D molecular structures were overlapped using atom-centered Gaussians [45]. A bias on the overlay was achieved by adding a positive weight to similar chemical groups. For each template, the 100 best hits were selected (600 molecules overall).

The similarity in electrostatic potential between the selected molecules and their templates in Figure 1 was then calculated using the Tanimoto metric [32]. The EON module of Openeye was used (See Supporting Information S1) [46]. The top 10 hits for each template (60 ligands overall) were docked onto 6 α-syn representative conformations of the protein NMR conformational ensemble; the same procedure as described previously by Herrera *et al*. [29]. The adducts were ranked based on the number of contacts [29] between the target region ^125^YEMPS^129^ [23], [25], [27] and the ligand. Five commercially available ligands forming top ranking adducts were selected for experimental analysis (Figure 2).

### MD Simulations of Ligands (i) and (ii) to α-Syn

All of the adducts, with all the five ligands, underwent 6 ns of MD in explicit solvent, as previously reported [29]. Version 2.6 of the NAMD program was used [47]. Overall, 72 different MD simulations were carried out. We identify as bound complexes the adducts in which the distance between Cα of α-syn and ligands center of mass is lower than 8 Å.

### Expression, Purification and Characterization of Recombinant Human α-Syn

All chemicals used for the experimental assays were high-grade analytical chemicals >99.9% purity with minimal metal impurities.

Human α-syn nucleotide sequence was cloned into the pET11a expression vector and introduced into *E. coli* strain BL21 (DE3). Expression of α-syn was obtained by growing cells in 100 µg/mL ampicillin containing Luria-Bertani broth at 37°C until an OD600 of about 0.6, followed by induction with 0.6 mM isopropyl β-thiogalactopyranoside for 5 hours. The protein was purified, based on the method of Huang *et al*. [34].

### Mass Spectrometry

The exact molecular weight of the purified α-syn was analyzed by reverse phase high performance liquid chromatography (HPLC) followed by mass spectrometry. Reverse phase HPLC was carried out using a C4 Phenomenex Jupiter (150×4.6 mm) and eluted with a gradient of acetonitrile and trifluoroacetic acid (TFA) 0.085% versus water and 0.1% TFA: from 5 to 35% over 5 min, from 35 to 55% over 20 min, from 55 to 95% over 2 min, at a flow rate of 0.6 mL/min. The effluent from the column was monitored by recording absorbance at 226 nm wavelength. The eluted peak was collected and analyzed by mass spectrometry using a Mariner System 5220 (Applied Biosystem) spectrometer. Mass measurements were carried out in collaboration with Dr. P. De Laureto (C.R.I.B.I., University of Padua, Italy).

### Circular Dichroism (CD) Spectroscopy

CD measurements were carried out on a JASCO J-810 spectrophotometer. Spectra were acquired at room temperature in Tris-HCl 20 mM, pH 8.0 using a HELLMA quartz cell (Cell Bio), with an optical path-length of 0.1 cm. All spectra were recorded in the 196–250 nm wavelength range, using a bandwidth of 1 nm and a time constant of 1 s at a speed of 20 nm/min. The signal-to-noise ratio was improved by accumulating 4 scans.

### Preparation of α-Syn Solutions and Dopamine Analogs for the *In Vitro* Studies

The solutions for the *in vitro* assay were prepared as follows. All solutions were sterile, filtered through a 0.22 µm filter prior to each assay run in order to reduce the presence of ‘contaminants’. Lyophilized α-syn was dissolved in 20 mM Tris-HCl/150 mM NaCl/pH 7.4 (*i.e.*: the fibrillization buffer) to achieve a stock concentration of 3 mg/mL. All 5 test ligands (Figure 2) and dopamine were dissolved in DMSO, to achieve a final stock concentration of 10 mM. The ligands and α-syn were then both diluted with the fibrillization buffer, to equimolar concentrations (100 µM) in a final DMSO concentration of 0.1%. Amyloid fibril formation was monitored with the histological dye, ThT. A total of 10 µM ThT was added to each sample. Final working volumes were 200 µL per well. Fibril formation was monitored on either a GEMINI EM plate reader or a Spectramax M5 (Molecular Devices). Each test ligand with α-syn was run in triplicate, in 96 black well plates; each well containing 1 teflon bead. The plate was incubated at 37°C, shaken, and ThT fluorescence readings were recorded every 5 min till 100 hours. For each assay run, the background fluorescence from each ligand (*i.e.* in the absence of α-syn) was also recorded and run in triplicate.

### AFM Analysis

The assay end product was imaged with a NanoWizard-II BioAFM (JPK Instruments AG) operating in dynamic mode. Scans were made using an ARROW™ silicon probe with Al coating at the detector side, a tip radius <10 nm, a nominal spring constant of 42 N/m and a nominal resonance frequency of 285 kHz (NanoWorld). Fibrils were deposited onto a freshly cleaved piece of mica at a concentration of 15 µM and left to adhere for 60 min. Samples were then washed with distilled water and blow-dried under a flow of nitrogen. Optically clear regions were chosen for the scanning analysis. The images were collected at a line scan rate of 0.5 - 2 Hz in ambient conditions. The AFM free oscillation amplitudes were ranging from 25 nm to 40 nm, with characteristic set points ranging from 75% to 90% of these free oscillation amplitudes.

### TEM Analysis

The assay end product was analyzed on a Jeol 2010F UHR TEM/STEM microscope operated at an accelerating voltage of 200 kV. Samples were absorbed to 300 µm holey formvar/carbon coated grids for ≈1 minute before a brief rinse in water, and negatively stained for 1 minute with 1% phosphotungstic acid. Typical magnifications ranged from 20000-180000.

### Image Analysis of AFM Scans and TEM Micrographs

AFM data were analyzed with Gwyddion (gwyddion.net) and SPIP™, (www.imagemet.com). TEM images were analyzed, using the NIH Image processing program, Image J (rsbweb.nih.gov/ij/).

## Supporting Information

Supporting Information S1 Supporting information manuscript including references.
(0.04 MB DOC)

Table S1 Molecules selected from the ligand.info database. Ten molecules have been selected for each of the six compounds reported in Figure 1. These are the molecules which feature the largest shape and electrostatic similarity with dopamine, as calculated using the Tanimoto's definition. The compounds for each set are listed in the order of the priority score. Five commercially available ligands among these 60 molecules have undergone the in vitro assay reported in this study. They are highlighted in bold.
(0.06 MB DOC)

Table S2 Hydrogen bonds and hydrophobic contacts between the ligands (as depicted in Figure 2) or the dopamine oxidation products (Figure 1) and the target region of α-syn conformations. Column titles from left to right: (i) number of the representative conformation of α-syn, (ii) ligand name, (iii) hydrogen-bonds and distances, (iv) hydrophobic contacts and distances. The contacts are listed for the adducts where the ligands are bound to the target region for more than 50% of their time. Highlighted in grey are the compounds used in the experiments.
(0.10 MB DOC)

Table S3 Binding regions of ligands other than the target region. Column titles from left to right: (i) The representative conformation of α-syn, (ii) ligand name, (iii) binding region. The contacts are listed for those adducts in which the ligands bind to regions other than the target region for more than 50% of their time. Highlighted in grey are the compounds used in the experiments.
(0.05 MB DOC)

Figure S1 The average lag phase time of Î±-syn aggregation in different concentrations of DMSO. Notice that above 2.5% DMSO concentration, the lag phase time becomes extremely variable.
(0.45 MB TIF)

Figure S2 AFM images of the aggregation of Î±-syn. AFM height images were acquired from assay end products after 100 hours of incubation which had been deposited onto freshly cleaved mica surfaces. The height images (A, B, D) are displayed using the same color scale as shown on the right hand side. (A) Height image of long straight fibrils with occasional ‘putative’ looking oligomers. (B) Height image and (C) phase image (range 0–13 deg) of a cluster of fibrils. (D) Height image showing a high degree of clustering of fibrils.
(3.15 MB TIF)

Figure S3 AFM images of the aggregation of Î±-syn in 0.1% DMSO. AFM height images were acquired from assay end products after 100 hours of incubation which had been deposited onto freshly cleaved mica surfaces. The height images (A, C, D) are displayed using the same color scale as shown on the right hand side. (A) Height image and (B) phase image (range: 0–40 deg) of long straight fibrils with branching. (C–D) Height images of long, straight and circular fibrils, fragments, and both clustered and scattered ‘putative’ oligomers.
(3.18 MB TIF)
