# Supplementary material for: Modulation of Alpha-Synuclein Aggregation by Dopamine Analogs
Source: PLoS One. 2010 Feb 16;5(2):e9234. doi: 10.1371/journal.pone.0009234 (PMC2821914; doi:10.1371/journal.pone.0009234)
Supplement: Supporting Information S1 — Supporting information manuscript including references. (0.04 MB DOC) [file pone.0009234.s001.doc]

**Modulation of alpha-synuclein aggregation by dopamine analogs**

**SUPPORTING INFORMATION**

**EON.**

The EON program ([www.eyesopen.com](http://www.eyesopen.com/)) calculates the electrostatic potential around each molecule using the ZAP toolkit. ZAP the Poisson–Boltzmann equation wherein molecular interiors are assigned one dielectric constant, and exteriors (typically water) another, thereby taking into account solvent polarization. Potentials are here extended out from the molecular surface by 6 Å or more and are enumerated on a regular lattice, or grid, with a spacing of about 1 Å. Once the potential is calculated, it uses a field-base measure of Tanimoto (Tanimoto, 1957), to compare the electrostatic potential of two molecules. The values for the Tanimoto score range from 1 (identical) to negative values resulting from the overlap of positive and negative charges (Nicholls et al, 2004).

The atomic charges were calculated at the AM1-BCC semi-empirical level using the MOLCHARGE module of OpenEye ([www.eyesopen.com](http://www.eyesopen.com/)).

**Docking.**

The resulting 70 molecules were docked onto 6 alpha-synuclein (α-syn) representative conformations using AUTODOCK 3.1 (Morris, 1998). Since α-syn is unstructured, a set of 6 conformations representing about 75% of the total number of conformers were selected from the NMR ensemble of 4000 structures using a cluster methodology as described in Herrera *et al.* (Herrera et al, 2008).

The Lamarckian genetic algorithm was applied to identify low energy binding sites and orientations of dopamine (DOP) mimics. Amber atom charges were assigned to the protein and AM1-BCC charges to the ligand atoms. The potential grid map for each atom type was calculated using a cubic box of 252 grid points in each direction, with a distance of 0.5 Å between grid points. For each complex, 100 docking runs were performed resulting in a total of 42000 calculations.

The 70 adducts were scored based on the number of contacts between the target region ^125^YEMPS^129^ and the ligand. A contact is considered when the distance between the alpha carbon (Cα) of α-syn residues and the centre of the mass of the ligand is less than 5 Å. Among them five commercially available molecules were selected for further molecular dynamics (MD) simulations and *in vitro* studies (Table S1).

**Molecular dynamics (MD)**

Each of the six α-syn representatives, forming a complex with each of the five commercially available molecules, underwent 6 ns of MD in explicit solvent. The RESP atomic charges of each ligand were calculated at the HF/6-31G* level of theory, using the GAUSSIAN98 suite of programs . Each complex was inserted into a box of ~15000 water and the overall charges of the system was neutralized by adding 9 Na^+^ counter ions. Periodic boundary conditions were applied, taking care that the minimum distance between α-syn and its images was larger than 12 Å. The AMBER99 force field (Case et al, 2005) was used for the macromolecule and counter ions, and the TIP3P force field (Jorgensen et al, 1983) was used for water molecules. Electrostatic interactions were calculated using the particle mesh Ewald method. A cutoff distance of 10 Å for the real part of the electrostatic and van der Waals interactions was used. The time-step was set to 2 fs. The SHAKE algorithm was applied to fix all bond lengths. The simulations were performed at T = 300 K and P = 1.013 bar by coupling the systems with a Langevin thermostat with a coupling coefficient of 5 ps, and a Nosè-Hoover Langevin barostat, with an oscillation period of 200 fs and the damping timescale of 100 fs. The pressure coupling allowed the system to reach a water density of about 0.98 g/cc. All the MD simulations were performed using the NAMD 2.6 program (Phillips et al, 2005).

**Electrostatic interactions**

According to a simple and very approximate electrostatic calculation that uses RESP atomic charges E83 is the NAC residue forming the largest long-range electrostatic stabilization of DOP adducts. We used the same point charge model *in vacuo*, assuming a dielectric constant of 1 to calculate the electrostatic interaction energies between E83 and the ligands. For each ligand we calculated the percentage of a total simulated time (36 ns) during which the ligand was present within 12 Å from Cα of the E83.

**SUPPORTING INFORMATION REFERENCES**

Tanimoto T (1957) IBM Internal Report.

Nicholls A, MacCuish NE, MacCuish JD. Variable selection and model validation of 2D and 3D molecular descriptors. J Comput Aided Mol Des 2004 Jul-Sep; 18 (7-9): 451-74.

Morris, G. M. (1998) in *Department of Molecular Biology*, ed. Laboratory, M. G. (The Scripps Research Institute, San Diego, CA, USA).

Herrera, F. E., Chesi, A., Paleologou, K. E., Schmid, A., Munoz, A., Vendruscolo, M., Gustincich, S., Lashuel, H. A. & Carloni, P. (2008) *PLoS ONE* **3,** e3394.

Case, D. A., Cheatham, T. E., 3rd, Darden, T., Gohlke, H., Luo, R., Merz, K. M., Jr., Onufriev, A., Simmerling, C., Wang, B. & Woods, R. J. (2005) *J Comput Chem* **26,** 1668-88.

Jorgensen, W. L., Chandrasekhar, J., Madura, J. D., Impey, R. W. & Klein, M. L. (1983) *J Chem Phys***,** 926-35.

Phillips, J. C., Braun, R., Wang, W., Gumbart, J., Tajkhorshid, E., Villa, E., Chipot, C., Skeel, R. D., Kale, L. & Schulten, K. (2005) *J Comput Chem* **26,** 1781-802.
