# Supplementary material for: Modulation of Alpha-Synuclein Aggregation by Dopamine Analogs
Source: PLoS One. 2010 Feb 16;5(2):e9234. doi: 10.1371/journal.pone.0009234 (PMC2821914; doi:10.1371/journal.pone.0009234)
Supplement: Table S1 — Molecules selected from the ligand.info database. Ten molecules have been selected for each of the six compounds reported in Figure 1. These are the molecules which feature the largest shape and electrostatic similarity with dopamine, as calculated using the Tanimoto's definition. The compounds for each set are listed in the order of the priority score. Five commercially available ligands among these 60 molecules have undergone the in vitro assay reported in this study. They are highlighted in bold. (0.06 MB DOC) [file pone.0009234.s002.doc]

**Table S1.** **Molecules selected from the *ligand.info* database.** Ten molecules have been selected for each of the six compounds reported in Figure 1. These are the molecules which feature the largest shape and electrostatic similarity with dopamine, as calculated using the Tanimoto’s definition. The compounds for each set are listed in the order of the priority score. Five commercially available ligands among these 60 molecules have undergone the *in vitro* assay reported in this study. They are highlighted in bold.

| DOPH | 2-(3,4-dihydroxyphenyl)ethyl-trimethylazanium |
| --- | --- |
|  | 3-hydroxy-1-(2-hydroxyethyl)-2-methylpyridin-4-one |
|  | 1-(2-aminoethyl)-3-hydroxy-2-methylpyridin-4-one |
|  | 6-(1,2-dihydroxyethyl)-3,4-dihydro-2H-pyran-2,3,4-triol |
|  | N-(2,3,4,6-Tetrahydroxy-5-oxo-cyclohexyl)-guanidine |
|  | 5-(2-aminoethyl)benzene-1,2,4-triol |
|  | 4-(2-amino-1-hydroxyethyl)benzene-1,2-diol |
|  | 2-Amino-3-(3,4-dihydroxy-phenyl)-2-methyl-propionic |
|  | 5-(2-aminoethyl)benzene-1,2,3-triol |
|  | 4-(2-amino-1-hydroxyethyl)-5-nitrobenzene-1,2-diol |
| DOP | 4-(2-methylaminoethyl)benzene-1,2-diol |
|  | 4-(2-aminoethyl)-2-methoxyphenol |
|  | **4-(2-aminoethyl)phenol** |
|  | 4-(2-aminoethyl)-2-(hydroxymethyl)phenol |
|  | **4-(2-aminoethyl)aniline** |
|  | 4-(2-methylaminoethyl)phenol |
|  | 2-amino-4-propan-2-ylphenol |
|  | 4-propylbenzene-1,2-diol |
|  | **2-amino-4-tert-butylphenol** |
|  | 3-(2-aminopropyl)phenol |
| DCH | 1H-indole-5,6-dione |
|  | 3-hydroxy-1-methyl-2,3-dihydroindole-5,6-dione |
|  | 5-hydroxy-2-methyl-1H-pyrazolo[4,3-e]pyrimidine-4,6-dione |
|  | 5,6-dioxo-2,4-dihydro-1H-indole-2-carboxylic acid |
|  | 5-amino-6-(hydroxymethyl)oxane-2,3,4-triol |
|  | 2-methoxy-7H-purin-6-amine |
|  | 3,5-diaminocyclohexane-1,2-diol |
|  | 2-Methoxy-9H-purin-6-ol |
|  | 6-Amino-9H-purin-2-ol |
|  | 2,3-bis(hydroxymethyl)-6-methoxy-1H-indole-4,7-dione |
| DHI | 2,3,4-trimethyl-1H-indole-5,6- |
|  | **1H-indol-5-ol** |
|  | 1-methyl-1,2,3,4-tetrahydroisoquinoline-6,7-diol |
|  | 5,6-dihydroxy-1H-indole-2-carboxylic acid |
|  | **1H-indol-6-amine** |
|  | 2-methyl-3,4-dihydro-1H-isoquinoline-6,7-diol |
|  | 2,4-diaminophenol |
|  | 6-methylbenzene-1,2,4-triol |
|  | 4-Amino-benzene-1,2-diol |
|  | 2-ethyl-3,4-dihydro-1H-isoquinoline-6,7-diol |
| IQ | 1H-Indole-5,6-dione |
|  | 1,3-Dimethyl-2,3-dihydro-1H-indole-5,6-dione |
|  | 2,3-Dihydro-1H-indene-5,6-dione |
|  | 1,3-Dimethyl-6-thioxo-1,2,3,6-tetrahydro-indol-5-one |
|  | 2-Isopropenyl-2,4-dihydro-1H-indene-5,6-dione |
|  | 2,3-Dihydro-benzofuran-5,6-dione |
|  | 1,3-Dimethyl-5-thioxo-1,2,3,5-tetrahydro-indol-6-one |
|  | 3-Fluoro-1H-quinolin-2-one |
|  | 5-Fluoro-1H-pyrrolo[2,3-c]pyridine |
|  | 3-Iodo-1H-quinolin-2-one |
| DQ | 4-(2-Amino-ethyl)-[1,2]benzoquinone |
|  | 4-(2-Hydroxy-ethyl)-[1,2]benzoquinone |
|  | 3-(2-Amino-ethyl)-6-thioxo-cyclohexa-2,4-dienone |
|  | 4-(2-Amino-ethyl)-6-thioxo-cyclohexa-2,4-dienone |
|  | 4-(2-Hydroxy-ethyl)-6-thioxo-cyclohexa-2,4-dienone |
|  | 5-(2-Amino-ethyl)-2-fluoro-thiophene-3-carboxylic acid amide |
|  | 3-(2-Hydroxy-ethyl)-6-thioxo-cyclohexa-2,4-dienone |
|  | 5-(2-Amino-ethyl)-2-iodo-thiophene-3-carboxylic acid amide |
|  | 5-(2-Amino-ethyl)-2-fluoro-furan-3-carboxylic acid amide |
|  | 3-Fluoro-1-(2-hydroxy-ethyl)-2-methyl-1H-pyridin-4-one |
