# Supplementary material for: Modulation of Alpha-Synuclein Aggregation by Dopamine Analogs
Source: PLoS One. 2010 Feb 16;5(2):e9234. doi: 10.1371/journal.pone.0009234 (PMC2821914; doi:10.1371/journal.pone.0009234)
Supplement: Table S2 — Hydrogen bonds and hydrophobic contacts between the ligands (as depicted in Figure 2) or the dopamine oxidation products (Figure 1) and the target region of α-syn conformations. Column titles from left to right: (i) number of the representative conformation of α-syn, (ii) ligand name, (iii) hydrogen-bonds and distances, (iv) hydrophobic contacts and distances. The contacts are listed for the adducts where the ligands are bound to the target region for more than 50% of their time. Highlighted in grey are the compounds used in the experiments. (0.10 MB DOC) [file pone.0009234.s003.doc]

**Table S2. Hydrogen bonds and hydrophobic contacts between the ligands (as depicted in Figure 2) or the dopamine oxidation products (Figure 1) and the target region of α-syn conformations.** Column titles from left to right: (i) number of the representative conformation of α-syn, (ii) ligand name, (iii) hydrogen-bonds and distances, (iv) hydrophobic contacts and distances. The contacts are listed for the adducts where the ligands are bound to the target region for more than 50% of their time. Highlighted in grey are the compounds used in the experiments.

|  | **Ligand name** | **Hydrogen bonds and distances (**Å**)*** | **Hydrophobic contacts and distances (**Å**)**^†^ |
| --- | --- | --- | --- |
| **01** | 6-aminoindole (KN1) | Tyr 125 (OH) – 6-aminoindole (N2) 3.81 ± 0.89 | Pro 120 6.43 ± 0.44  Ala 124 5.66 ± 0.41  Met 127 6.32 ± 0.50  Pro 128 6.88 ± 0.58  Gly 132 7.15 ± 0.50  Tyr 133 5.97 ± 0.63 |
|  | tyramine | Unstable | Unstable |
|  | 4-(2-aminoethyl)aniline |  | Gly 132 4.57 ± 1.05  Tyr 133 6.79 ± 0.96 |
|  | Dopamine | Unstable | Unstable |
|  | Indol-5,6-quinone | Unstable | Unstable |
|  | Dopamine-o-quinone (DQ) | Ser129 (O) – Dopamine-o-quinone (N1)  2.9 ± 0.95 | Gln 134 6.46 ± 0.92  Glu 136 6.63 ± 1.03 |
|  | Protonated dopamine | Glu 123 (OE1) – Protonated dopamine (O2), 3.3 ± 0.75 | Glu 131 4.12 ± 0.65  Gly 132 5.67 ± 0.69  Tyr 133 3.78 ± 0.81  Gln 134 4.66 ± 0.77 |
|  | | | |
| **02** | 5-hydroxyindole | Unstable | Unstable |
|  | tyramine | Unstable | Unstable |
|  | 2-amino-4-tert-butylphenol | Unstable | Unstable |
|  | 4-(2-aminoethyl)aniline | Unstable | Unstable |
|  | Dopamine | Asp 119 (N) – Dopamine (O1) (D=3.5 ± 0.8) | Met 116 6.18 ± 0.93  Val 118 5.03 ± 0.88  Tyr 125 5.14 ± 1.82 |
|  | Dopamine-o-quinone |  | Pro 120 6.13 ± 1.10  Tyr 125 5.45 ± 1.09 |
|  | Protonated dopamine | Unstable | Unstable |
|  | | | |
| **03** | 6-aminoindole | Unstable | Unstable |
|  | 5-hydroxyindole | Glu 126 (OE1) – 5-hydroxyindole (O1) 3.2 ± 0.97 | Met 116 5.35 ± 1.19  Pro 117 5.32 ± 1.09 |
|  | tyramine | Glu 109 (OE1) – tyramine (O1) 2.9 ± 1.02 | Tyr 125 5.54 ± 1.12 |
|  | 2-amino-4-tert butylphenol | Gln 134 (O) - 2-amino-4-tert butylphenol (N1) 3.6 ± 0.71 | Tyr 125 6.77 ± 0.91  Glu 126 6.16 ± 1.32  Met 127 5.64 ± 1.09 |
|  | 4-(2-aminoethyl)aniline | Unstable | Unstable |
|  | Dopamine | Unstable | Unstable |
|  | Dopamine-o-quinone |  | Met 116 6.61 ± 1.31  Pro 117 6.21 ± 1.05  Val 118 5.95 ± 0.79  Tyr 125 5.71 ± 0.54  Glu 126 6.69 ± 0.88  Met 127 6.15 ± 0.72  Tyr 133 5.29 ± 1.25 |
|  | Protonated dopamine |  | Glu 123 6.03 ± 1.15  Ala 124 5.33 ± 0.95  Tyr 125 6.84 ± 1.06  Glu 126 6.66 ± 1.07 |
|  | Dopaminochrome | Ser 129 (OG) – Dopaminochrome (O2)  3.55 ± 0.41 | Met 127 6.29 ± 0.97  Pro 128 7.15 ± 0.89  Glu 131 5.87 ± 1.20  Gly 132 6.08 ± 1.59  Tyr 136 5.29 ± 0.95 |
|  | Indol-5,6-quinone | Ser 129 (O) – Indol-5,6-quinone (O2)  3.02 ± 0.64 | Pro 128 5.57 ± 1.09 |
|  | | | |
| **04** | tyramine |  | Met 127 5.62 ± 1.10  Gly 132 6.09 ± 1.45  Tyr 133 5.47 ± 1.92 |
|  | 2-amino-4-tert-butylphenol |  | Val 66 5.63 ± 0.73  Tyr 125 4.66 ± 0.56  Glu 126 5.21 ± 0.61 |
|  | 4-(2-aminoethyl)aniline | Unstable | Unstable |
|  | Dopamine | Glu 131 (OE1) – Dopamine (O1), 3.35 ± 0.54 | Glu 130 6.75 ± 0.98  Gly 132 5.91 ± 0.88  Tyr 133 6.57 ± 1.11 |
|  | Dopamine-o-quinone | Unstable | Unstable |
|  | Protonated dopamine | Ser 129 (O) – Protonated dopamine (N1) 3.77 ± 0.82 | Glu 130 6.96 ± 1.13 |
|  | Dopaminochrome | Unstable | Unstable |
|  | Indol-5,6-quinone | Unstable | Unstable |
|  | | | |
| **05** | 6-aminoindole | Unstable | Unstable |
|  | 5-hydroxyindole |  | Tyr 125 6.00 ± 0.86  Glu 126 4.67 ± 0.74  Met 127 5.81 ± 0.86 |
|  | tyramine | Unstable | Unstable |
|  | 2-amino-4-tert-butylphenol | Unstable | Unstable |
|  | 4-(2-aminoethyl)aniline | Unstable | Unstable |
|  | Dopamine | Glu 105 (OE1) – Dopamine (O1), 3.56 ± 1.10 | Glu 123 6.77 ± 1.15 |
|  | 5,6-dihydroxyindole | Gln 109 (OE1) – Dihydroxyindole (N1),  3.30 ± 0.98 | Glu 123 5.93 ± 1.06  Ala 124 6.05 ± 1.10  Tyr 125 5.96 ± 1.08 |
|  | Dopamine-o-quinone | Unstable | Unstable |
|  | Dopaminochrome |  | Tyr 125 4.75 ± 1.14 |
|  | Indol-5,6-quinone |  | Ala 124 6.19 ± 1.25  Tyr 125 4.92 ± 1.05 |
|  | | | |
| **06** | 5-hydroxyindole | Unstable | Unstable |
|  | Dopamine | Unstable | Unstable |
|  | Dopamine-o-quinone | Unstable | Unstable |
|  | Dopaminochrome | Unstable | Unstable |

^*^ Distance is measured between the heavy atoms.

^†^ Distance is measured between the center of mass of the ligand and the specific amino acid.
