# Supplementary material for: Modulation of Alpha-Synuclein Aggregation by Dopamine Analogs
Source: PLoS One. 2010 Feb 16;5(2):e9234. doi: 10.1371/journal.pone.0009234 (PMC2821914; doi:10.1371/journal.pone.0009234)
Supplement: Table S3 — Binding regions of ligands other than the target region. Column titles from left to right: (i) The representative conformation of α-syn, (ii) ligand name, (iii) binding region. The contacts are listed for those adducts in which the ligands bind to regions other than the target region for more than 50% of their time. Highlighted in grey are the compounds used in the experiments. (0.05 MB DOC) [file pone.0009234.s004.doc]

**Table S3. Binding regions of ligands other than the target region.** Column titles from left to right: (i) The representative conformation of α-syn, (ii) ligand name, (iii) binding region. The contacts are listed for those adducts in which the ligands bind to regions other than the target region for more than 50% of their time. Highlighted in grey are the compounds used in the experiments.

| **Conformation** | **Ligand name** | **Binding region (residue number)** |
| --- | --- | --- |
| **01** | 5-hydroxyindole | 38 to 45 |
|  | 2-amino-4-tert-butylphenol | 65 to 80 |
|  | 5,6-Dihydroxyindole | 66 to 69 |
|  | Dopaminochrome | 62 to 77 |
|  | | |
| **02** | 6-aminoindole | 103 to 110 |
|  | Indol-5,6-quinone | 58 to 69 |
|  | 5,6-Dihydroxyindole | 93 to 101 |
|  | Dopaminochrome | 94 to 100 |
|  | | |
| **03** | 5,6-Dihydroxyindole | 38 to 45 |
|  | | |
| **04** | 6-aminoindole | 49 to 77 |
|  | 5-hydroxyindole | 56 to 77 |
|  | 2-amino-4-tert-butylphenol | 53 to 78 |
|  | 5,6-dihydroxyindole | 59 to 69 |
|  | | |
| **05** | Protonated dopamine | 112 to 119 |
|  | | |
| **06** | 6-aminoindole | 71 to 75 |
|  | tyramine | 55 to 75 |
|  | 2-amino-4-tert-butylphenol | 64 to 80 |
|  | 4-(2-aminoethyl)aniline | 63 to 70 |
|  | 5,6-dihydroxyindole | 51 to 71 |
|  | Protonated dopamine | 57 to 72 |
|  | Indol-5,6-quinone | 62 to 77 |
